# AI-assisted transcription of YouTube videos on penile enlargement: analysis of their text quality and readability

**DOI:** 10.1093/sexmed/qfaf023

**Published:** 2025-04-21

**Authors:** Mehmet Fatih Şahin, Erdem Can Topkaç, Çağrı Doğan, Serkan Şeramet, Furkan Batuhan Tuncer, Muhammed Sencer Köroğlu, Onur Orbeği, Cenk Murat Yazici

**Affiliations:** Faculty of Medicine Department of Urology, Tekirdağ Namık Kemal University, Süleymanpaşa, Tekirdağ 59020, Turkey; Faculty of Medicine Department of Urology, Tekirdağ Namık Kemal University, Süleymanpaşa, Tekirdağ 59020, Turkey; Faculty of Medicine Department of Urology, Tekirdağ Namık Kemal University, Süleymanpaşa, Tekirdağ 59020, Turkey; Faculty of Medicine Department of Urology, Tekirdağ Namık Kemal University, Süleymanpaşa, Tekirdağ 59020, Turkey; Faculty of Medicine Department of Urology, Tekirdağ Namık Kemal University, Süleymanpaşa, Tekirdağ 59020, Turkey; Faculty of Medicine Department of Urology, Tekirdağ Namık Kemal University, Süleymanpaşa, Tekirdağ 59020, Turkey; Faculty of Medicine Department of Urology, Tekirdağ Namık Kemal University, Süleymanpaşa, Tekirdağ 59020, Turkey; Faculty of Medicine Department of Urology, Tekirdağ Namık Kemal University, Süleymanpaşa, Tekirdağ 59020, Turkey

**Keywords:** YouTube, penile enlargement, whisperAI, transcription, artificial intelligence

## Abstract

**Background:**

Patients dealing with sensitive issues like penile enlargement (PE) might benefit from YouTube videos. Therefore, it is essential that the textual content of these videos is clear, trustworthy, and of high quality.

**Aim:**

Are the AI-assisted acquired texts’ qualities and comprehensibilities of YouTube videos about PE enough and suitable for the patients?

**Methods:**

On October 25, 2024, Google Trends analysis identified the 25 most searched phrases for “Penile enlargement.” Non-related terms were excluded, and the relevant keywords were searched on YouTube. Only content about PE included; excluding duplicates, non-English videos, YouTube shorts, those under 30 seconds, silent, and music-only videos. Videos were transcribed using Whisper AI, and their quality was assessed by M.F.Ş, E.C.T., and Ç.D. using the GQS (global quality scale) and DISCERN, the readability was evaluated via Flesch–Kincaid (FKGL and FKRE) measures. High assessor agreement was noted (Pearson r = 0.912). Videos were categorized by uploader, and metrics such as views, likes, comments, and duration were recorded. The Chi-square test was used for categorical variable comparisons; the Kruskal-Wallis H-Test was applied when normality and homoscedasticity were not met, with Bonferroni post hoc correction for multiple comparisons.

**Outcomes:**

The mean DISCERN and GQS scores were 51.23 ± 13.1 and 3.32 ± 0.9, respectively. FKRE and FKGL scores were 73.12 ± 11.7 and 5.85 ± 2.1. Physicians (n = 67) produced the most videos, while academic institutions (n = 2) produced the least. No significant differences in text quality were found between groups (*P* = 0.067 and *P* = 0.051). Health-related websites exhibited lower FKRE compared to non-healthcare videos (*P* = **0.002**), with a significant difference in FKGL as well (*P* = **0.019**).

**Results:**

The video exhibited a high level of readability (indicating comprehensibility for almost a 6th-grade student). Text quality, view and like count of the videos uploaded by academic institutions was the highest.

**Clinical Implications:**

In PE, YouTube video’s health information needs to be better quality and more trustworthy, according to our research. The language used in videos should be easier to understand.

**Strengths and Limitations:**

This study is the first scientific analysis of YouTube video transcripts on PE using AI, focusing specifically on English content, which limits its applicability to non-English speakers and other platforms. Exclusions of silent and shorter videos may result in the omission of valuable information.

**Conclusion:**

The need for better quality and trustworthiness in health-related YouTube information, especially for PE is essential. Content makers should stress clear, accessible language and minimize disinformation.

## Introduction

Penile enlargement (PE) remains a significant subject of interest within the field of andrology. Men have historically expressed concern regarding the sizes of their penises and often seek to enhance size as a means of boosting self-esteem or impressing and satisfying their partners.^1^ Anxiety related to genital size can impact men’s sexual functionality and pleasure, driving them to explore both surgical and non-surgical PE options.^2^ Recently, there has been a marked increase in the demand for PE procedures. Currently, the only widely accepted indications for such interventions pertain to the surgical correction of conditions such as micropenis and Peyronie’s disease.^3^ There are also publications indicating that penile length shortens after radical prostatectomy operations.^4^ Despite the growing number of surgical and non-invasive techniques available and an increasing volume of literature on the topic, patients often seek information regarding these approaches’ effectiveness and comparative merits. Consequently, many individuals turn to various online platforms to address their inquiries and concerns.

YouTube is one of the most widely used digital communication platforms, hosting ~30 million health-related videos daily and amassing billions of videos overall. However, most of these videos are uploaded by unverified accounts and lack peer review.^5^ YouTube is a crucial social media platform for disseminating health-related information, utilizing videos to convey complex medical concepts engagingly, and enhancing comprehension and retention.^6^ The platform’s broad and diverse user demographic enables health professionals and organizations to reach a substantial audience, increasing awareness of health issues.^7^ Assessing the quality and usefulness of health-related videos on YouTube is paramount. In educating patients, the clarity and quality of the verbal information presented are significantly more critical than the visual elements included in the videos. Given the overwhelming volume of health content on the site, careful evaluation is essential to ensure authenticity and reliability, safeguarding visitors from misleading or potentially harmful information.^8^ Furthermore, research provides essential insights that can inform guidelines and best practices, ultimately enhancing the quality of health content on YouTube and improving public health awareness.^9^

While existing literature includes studies assessing Youtube video quality about PE,^10^ there has been a lack of examination concerning the quality and readability of the textual content. This study aims to evaluate the quality and readability of the texts derived from the AI transcription of YouTube videos. Are the quality and comprehensibility of AI-assisted texts derived from YouTube videos on PE enough and appropriate for patients?

## Materials and methods

The study was performed on 25th October 2024 at the urology department of Tekirdag Namik Kemal University. Ethics committee approval is unnecessary since no human-related material was used. To reduce the influence of user activity, the search was performed in incognito mode on Google Chrome after deleting the search history. A detailed compilation of the 25 most often searched terms for Google Trends was created with the keyword “Penile enlargement,” including a wide range of topics in Google’s online search inquiries on YouTube. Four words were excluded from the research owing to their irrelevance to the topic or their shortness and incompleteness: “Breast enlargement,” “Breast,” “Penis enlargement pills clown,” and “Penis enlargement in Hindi.” Subregions were used to categorize and document the geographically significant locations. Five hundred twenty-five videos (top 25 videos for each search keyword) were documented from the first page of YouTube search results. All chosen videos were archived in an Excel spreadsheet to facilitate a thorough study since YouTube search results may change. The inclusion criteria were videos primarily focused on PE while excluding unrelated videos, duplicates, non-English videos, YouTube short videos, videos shorter than 30 seconds, those devoid of speaking, and videos with only music according to established criteria, in alignment with prior research.^11^ Following the application of the exclusion criteria, the final sample consisted of 155 videos for further analysis.

### Readability assessment

An automatic voice recognition and transcription program, Whisper AI, was used to evaluate the comprehensibility of the videos.^12^ Whisper AI uses artificial intelligence (AI) to transcribe spoken words into written text. The resultant transcripts were further analyzed using the Flesch–Kincaid Reading Ease (FKRE) and Flesch–Kincaid Grade Level (FKGL) metrics to ascertain the text’s readability levels. The FKRE algorithm evaluates a document’s readability by analyzing the average sentence length and number of syllables per word. It computes the disparity between the two products and deducts it from 206.835 to ascertain the reading ease score. Elevated scores indicate more accessible content, whereas a score below 30 implies a reading ability equivalent to that of a college graduate.^13^ The FKGL algorithm assesses the educational grade level necessary to comprehend a text by analyzing sentence length and syllable count. The process entails many calculations: dividing the total number of words by the total number of sentences, multiplying the outcome by 0.39, dividing the total syllables by the total words, multiplying that result by 11.8, summing the derived figures, and finally subtracting 15.59 from the overall result. A lower grade level score signifies simpler understanding, while a higher score implies more intricate language.^14^

### Quality assessment

The Global Quality Scale (GQS) is a five-point measure used by investigators to evaluate videos based on ease of use, audiovisual quality, and intelligibility. Those scoring 1-2 are deemed poor quality; those scoring 3 are categorized as intermediate quality, while those scoring 4-5 are labeled excellent.^15^ The accuracy assessment of the data in each passage was performed with the DISCERN questionnaire, a validated tool designed to aid information providers and patients in evaluating the quality of written material on treatment options. The questionnaire seeks to facilitate the development of credible and scientifically validated health information for consumers by providing standards and serving as a guide for authors. The tool has 15 questions and enables evaluation on a scale from 1 to 5.^16^ M.F.Ş., Ç.D., and E.C.T., interested in andrology, executed the assessment processes for GQS and DISCERN. The association among assessors was powerful in evaluating the quality (Pearson correlation coefficient r = 0.912). In cases with differing opinions, a third expert with 20 years of experience in urology (C.Y.) served as an arbitrator to settle disputes and provide conclusions.

### Statistical analysis

The videos have been categorized by the uploader into six distinct categories: academia/universities, healthcare organizations, non-physician healthcare professionals, non-healthcare professionals, physicians, and health-related websites. The number of views, likes, comments, and length of time for all videos were also noted and compared. Measurements of central tendency and variability delineated the characteristics of quantitative variables: mean ± standard deviation. The Chi-square test was used to assess variations in proportions or associations among categorical variables. When the assumptions of normality and homoscedasticity were not met, the Kruskal-Wallis H-Test was applied to illustrate behavioral differences across group means. For multiple comparisons between groups, the Bonferroni post hoc correction was implemented for multiple group comparisons. Statistical significance was established at P = 0.05 for all instances. Statistical analyses were performed using the IBM SPSS (Statistical Package for the Social Sciences), version 29.0 (Armonk, NY, IBM Corp.)

## Results

### Keyword selection

**Figure 1:**
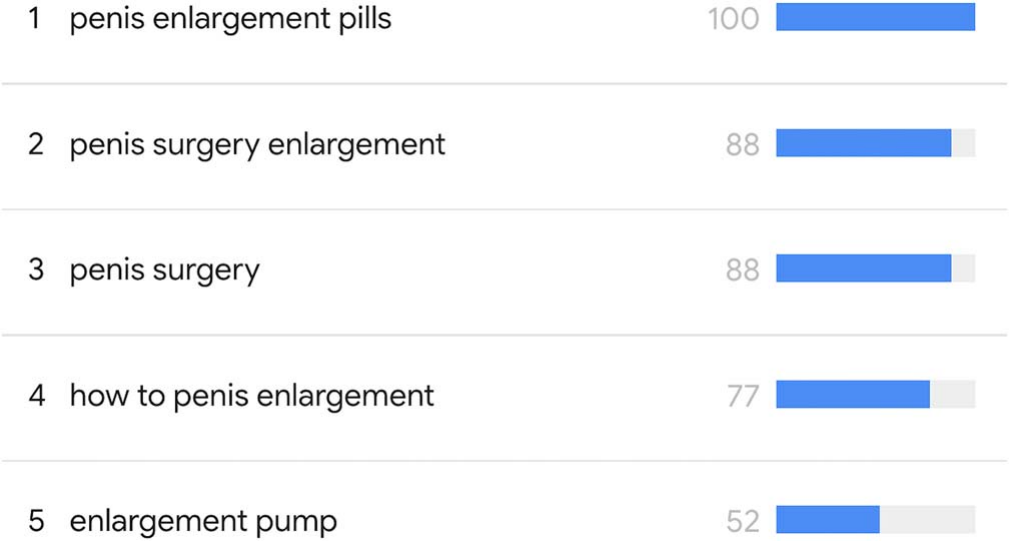
Top five keywords about PE.

The top five keywords were “penis enlargement pills”, “penis surgery enlargement”, “penis surgery”, “how to penis enlargement”, and “enlargement pump” (Figure 1). Four keywords were eliminated because they were irrelevant to the search (Table 1).

**Table 1:** Presents the Google Trends YouTube Search statistics for the top 25 phrases searched worldwide for penis enlargement in past 5 years.

**Rank**	**Keyword**	**Relevance**
**1**	Penis enlargement pills	100
**2**	Penis surgery enlargement	88
**3**	Penis surgery	88
**4**	How to penis enlargement	77
**5**	Enlargement pump	52
**6**	Penis enlargement exercise	43
**7**	Penis exercise	41
**8**	Natural penis enlargement	25
**9**	Penis increase	24
**10**	Penis enlargement medicine	23
**11**	Penis enlargement cream	21
**12**	Dick enlargement	20
**13**	Penis enlargement exercises	20
**14**	Penis exercises	20
**15**	Penis enlargement pills meme	13
**16**	How to make penis enlargement	12
**17**	Breast enlargement	12
**18**	Breast	12
**19**	Penile enlargement	12
**20**	Oil for penis enlargement	11
**21**	Penis enlargement pills clown	11
**22**	Penis enlargement in hindi	10
**23**	Increase penis size	10
**24**	How to increase penis	10
**25**	Penis enlargement treatment	9

### Quality and readability results

When all videos were evaluated, the average DISCERN and GQS scores were relatively low (51.23 ± 13.1 and 3.32 ± 0.9) and reasonably easy in terms of readability (FKRE score: 73.12 ± 11.7 and FKGL score: 5.85 ± 2.1, meaning an almost 6th-grade student can understand). Other results regarding the videos are shown in Table 2.

**Table 2:** Flesch–Kincaid Reading Ease, Flesch–Kincaid Grade Level, Global Quality Score, DISCERN, view count, length, number of likes and comment number of the videos.

	**Mean ± SD**	**Median (min-max)**
**DISCERN**	51.23 ± 13.1	51.0 (19-75)
**GQS**	3.32 ± 0.9	3.0 (1-5)
**FKRE**	73.12 ± 11.7	74.00 (29-98)
**FKGL**	5.85 ± 2.1	5.54 (1.1-12.3)
**View count number**	1118881.78 ± 2666582.8	279168.5 (125-30 813 457)
**Length (min)**	5.83 ± 7.1	4.15 (0.30-75.5)
**Number of Likes**	12142.77 ± 36278.7	1453.0 (0-420 106)
**Number of Comments**	656.94 ± 2348.5	102.0 (0-30 542)

### Comparison according to the uploader

When classified according to the uploader, the highest number of uploads among the videos were by physicians (n:67). The lowest was uploaded by academic institutions (n:2). It was found that there was no difference between the uploading groups in terms of the quality of the produced texts (*P* = 0.067 and *P* = 0.051) and that in terms of readability, the FKRE scores of the videos uploaded by health-related websites were statistically lower than the videos uploaded by non-healthcare professionals (*P* = **0.002**). A statistically significant difference was found between the groups regarding FKGL scores (*P* = **0.019**). No significant difference was found between the groups in terms of other characteristics of the videos (Table 3).

**Table 3:** Flesch–Kincaid Reading Ease, Flesch–Kincaid Grade Level, Global Quality Score, DISCERN, view count, length, total duration and like count of the videos according to the uploader.

	**Physicians**	**Health-related websites**	**Healthcare organizations**	**Non-healthcare professionals**	**Academia/Universities**	**Non-physician healthcare professionals**	**p**
**n, %**	67 (43.2%)	18 (11.6%)	22 (14.2%)	34 (21.9%)	2 (1.3%)	12 (7.8%)	
**DISCERN**	52.1 ± 14.2	42.9 ± 11.3	54.5 ± 9.2	51.5 ± 13.8	59.5 ± 16.3	50.8 ± 8.3	0.067^§^
	55.0 (48.6-55.5)	41.5 (37.2-48.5)	53.0 (50.4-58.6)	52.0 (46.7-56.3)	59.5 (-86.6-205.6)	50.5 (45.6-56.1)	
**GQS**	3.6 ± 0.9	2.8 ± 0.8	3.5 ± 0.5	3.2 ± 0.9	3.7 ± 0.7	3.2 ± 0.7	0.051^§^
	3.0 (3.2-3.7)	3.0 (2.4-3.2)	4.0 (3.4-3.8)	3.0 (2.9-3.5)	3.5 (-2.9-9.9)	3.0 (2.7-3.6)	
**FKRE**	72.5 ± 10.0	67.3 ± 14.7	72.1 ± 15.5	79.5 ± 9.1	67.0 ± 7.1	71.5 ± 8.0	**0.002** ^**1**^ ^**§**^
	73.0 (70.1-75.0)	72.0 (59.0-73.6)	74.0 (65.2-79.0)	79.5 (76.3-82.7)	67.0 (3.5-130.5)	71.0 (66.4-76.6)	
**FKGL**	5.9 ± 1.9	6.6 ± 2.5	6.1 ± 2.5	4.9 ± 2.1	6.7 ± 0.5	6.5 ± 1.3	**0.019** ^**1**^ ^**§**^
	5.5 (5.4-6.3)	6.0 (5.6-8.0)	5.5 (5.0-7.2)	4.9 (4.2-5.6)	6.7 (2.2-11.2)	6.5 (5.6-7.3)	
**View Count**	938312.1 ± 1641357.2	945316.7 ± 1485321.6	580207.7 ± 836811.0	1927846.9 ± 5262790.2	5279316.0 ± 7239724.0	1237281.3 ± 1329700.4	0.361^§^
	227320.0 (537953.7-1338670.6)	178187.5 (206683.7-1683949.8)	226797.0 (209186.5-951229.0)	476611.5 (91572.6-3764121.2)	5279316.0 (-59767113.2-70325745.2)	822546.0 (392430.0-2082132.7)	
**Total Duration**	5.1 ± 5.5	4.0 ± 3.7	10.5 ± 16.5	5.6 ± 4.3	4.3 ± 2.8	5.1 ± 3.7	0.347^§^
	3.5 (3.7-6.4)	3.2 (2.2-5.9)	5.9 (3.2-17.8)	4.3 (4.1-7.1)	4.3 (-21.2-29.9)	4.4 (2.8-7.5)	
**Like Count**	5185.6 ± 10619.4	8462.9 ± 19524.3	7150.2 ± 11368.3	23273.6 ± 72625.2	89004.5 ± 93360.0	17183.2 ± 27652.0	0.233^§^
	1368.0 (2595.3-7775.8)	866.0 (-1246.3-18172.1)	1121.0 (2109.8-12190.7)	2504.5 (-2066.5-48613.8)	89004.5 (-749802.0-927811.0)	5015.0 (-386.1-34752.4)	

(Mean ± SD/Median (Q1-Q3)

^1^: Differences are between videos of health-related websites and non-healthcare professionals.

§ : Kruskal-Wallis H-Test

## Discussion

This research evaluated the text quality, dependability, and comprehensibility of YouTube videos focused on PE. Most of these videos were designed for non-healthcare professionals, with simple scripts to read and comprehend. Moreover, most of the videos were considered beneficial and of above-average quality. The videos exhibited intermediate quality, a modest degree of trustworthiness, and, significantly, their comprehensibility above the specified standards. This study is the first scientific research to analyze YouTube video transcripts related to PE, rather than the overall video quality, using artificial intelligence.

Penile enlargement is a popular and delicate subject, with so many YouTube videos addressing it. These videos often showcase several methodologies, including medical interventions, physical activities, devices, and dietary supplements. Nonetheless, not all accessible information is trustworthy or supported by scientific data. At this stage, it is also essential who uploaded the video. Many videos are uploaded on internet platforms by physicians or healthcare professionals as well as by people who are not healthcare professionals and are not experts in their fields. These need to be distinguished, and the quality, understandability, and readability of the language used in the videos should be evaluated.

When we examined our study results, we found that the text quality of the videos uploaded by academic institutions was the highest and had the highest view and like count. This is due to the abundance of literature and the fact that they contain more academic information than other uploaders. For these reasons, the FKRE scores of these videos were found to be the lowest, meaning lower readability and FKGL scores were the highest, suggesting higher complexity. Videos uploaded by physicians came in second place in terms of quality. The quality of video texts created by professionals and trained people is indisputably higher. Healthcare specialists or reliable media sources may inform the patients about the advantages and disadvantages of performing medical treatments. Higher FKRE and lower FKGL scores may be ascribed to the strategic objective of academics and physicians’ content creators to convey information to a broader audience succinctly and directly.^17^ Consistent with several studies^18^^,^^19^ in the current literature, our results emphasize the need for doctors to have a more significant role in generating health-related material since their contributions attained the highest quality scores. This underscores the importance of medical practitioners in delivering dependable and credible health information.

Non-healthcare professionals’ videos mean FKRE scores were the highest, and the mean FKGL scores were the lowest; they had the easiest language to understand. According to our study, videos uploaded by health-related websites have the lowest quality. Although these videos are considered to have been uploaded from health-related websites, it is unclear who uploaded them. Therefore, it would be right to approach their content with suspicion. Many videos are aimed at telling people about techniques that are not supported by scientific evidence, as well as the use of miraculous therapies or unsafe ways, such as yoga. Some videos’ primary purpose is often to advertise goods or services rather than provide information. Those lacking expertise in specific fields frequently overstate, misrepresent, or oversimplify clear topics. For instance, claims of “guaranteed results” from a training program or “completely natural methods” in alternative medicine lack scientific support and can mislead the audience. These videos often rely on testimonials rather than referencing peer-reviewed research, which is commonly seen in their videos. They might use scientific terminology to create an impression of credibility. Additionally, many content creators enter into agreements with businesses or companies through affiliate relationships or sponsorships, promoting products like pills, pumps, or extenders without disclosing potential conflicts of interest. Often, people who do not have expertise pay attention only to technical problems that can be solved quickly and draw the viewers’ attention to immediate results. Practicing jelqing is a widely discussed technique for penis enlargement despite clinicians’ warnings about injuries and no efficacy.^20^ Indeed, they seldom mention the risks, for example, body injuries (scar, bruising, or nerve damage) and psychological effects such as increasing insecurities if the outcome is not pleasing. These situations require extreme caution in a sensitive issue such as PE, where the psychological background is also important.

Video is an excellent medical education medium, appropriate for both the general populace and healthcare practitioners.^21^ However, some studies have concluded that YouTube is not a very reliable source.^22^ When evaluating the potential instructional value of these videos for patient education, it is essential to proceed with care. Although these materials might enhance learning by offering insights into patient experiences, formal medical education is essential.^23^ Partnering with esteemed organizations can enhance credibility; nonetheless, it is imperative to remain vigilant in countering misinformation on social media platforms to guarantee that aspiring healthcare professionals, such as physicians focused on andrology, can access precise and reliable information.

Few studies in the current literature have explored the domain of YouTube video transcripts. Herbert et al.^24^ examined transcripts from YouTube videos related to pelvic organ prolapse in this specific field. Their results indicated that these transcripts were inconsistent with the prescribed educational level. Temel et al.’s analysis revealed that YouTube videos addressing traumatic brain injury surpassed the necessary grade level for understanding.^9^ Our study found that the text quality of the videos about PE was generally relatively low. This may be because, unlike the other diseases studied, PE is a subject that is open to much more abuse, and the videos are frequently watched on YouTube by patients other than healthcare professionals or experienced physicians.

This study has several limitations. The research only focused on English-language material accessible exclusively on YouTube. This limited scope may restrict its applicability to non-English-speaking populations (eg, Hindi or Chinese) and other video platforms. Furthermore, videos without audio, relatively shorter ones, were eliminated, possibly omitting crucial material. It is essential to use care when generalizing these results since social media usage may vary among countries owing to variables such as national identity, legitimacy concerns, interpersonal dynamics, and diplomatic difficulties. Although efficient, the study’s dependence on automated analytical techniques may fail to include video material and transcripts’ whole context and subtleties. There is a persistent need for research initiatives that cover a wider linguistic range, integrate material from other social media platforms, and provide a more comprehensive array of information.

## Conclusion

Our research reveals a pressing necessity for improved quality and reliability in YouTube health-related content, particularly in PE. Content creators should prioritize clear and accessible language while avoiding misinformation. Academics and healthcare professionals, as key contributors, can play a crucial role in bridging knowledge gaps for patients. Further research, regulations, educational initiatives, and potential platform oversight could work together to enhance the delivery of accurate health information on social media, ultimately benefiting content creators and users in this important field.

## Scientific responsibility statement

The authors declare that they are responsible for the article’s scientific content including study design, data collection, analysis and interpretation, writing, some of the main line, or all of the preparation and scientific review of the contents and approval of the final version of the article.
